# The identification of genes associated T-cell exhaustion and construction of prognostic signature to predict immunotherapy response in lung adenocarcinoma

**DOI:** 10.1038/s41598-023-40662-z

**Published:** 2023-08-17

**Authors:** Yahua Wu, Bin Du, Mingqiang Lin, Xiaohui Ji, Chengliu Lv, Jinhuo Lai

**Affiliations:** 1 Department of Medical Oncology, Fujian Medical University Union Hospital, No. 29 Xinquan Street, Fuzhou, 350000 Fujian China; 2 Department of Radiation Oncology, Fujian Medical University Cancer Hospital, Jin’an District, Fuzhou, 350000 Fujian China; 3 Department of Medical Oncology, Chongqing University Cancer Hospital, Chongqing, 400030 China

**Keywords:** Cancer, Genetics, Immunology

## Abstract

T-cell exhaustion (Tex) is considered to be a reason for immunotherapy resistance and poor prognosis in lung adenocarcinoma. Therefore, we used weighted correlation network analysis to identify Tex-related genes in the cancer genome atlas (TCGA). Unsupervised clustering approach based on Tex-related genes divided patients into cluster 1 and cluster 2. Then, we utilized random forest and the least absolute shrinkage and selection operator to identify nine key genes to construct a riskscore. Patients were classified as low or high-risk groups. The multivariate cox analysis showed the riskscore was an independent prognostic factor in TCGA and GSE72094 cohorts. Moreover, patients in cluster 2 with high riskscore had the worst prognosis. The immune response prediction analysis showed the low-risk group had higher immune, stromal, estimate scores, higher immunophenscore (IPS), and lower tumor immune dysfunction and exclusion score which suggested a better response to immune checkpoint inhibitors (ICIs) therapy in the low-risk group. In the meantime, we included two independent immunotherapy cohorts that also confirmed a better response to ICIs treatment in the low-risk group. Besides, we discovered differences in chemotherapy and targeted drug sensitivity between two groups. Finally, a nomogram was built to facilitate clinical decision making.

## Introduction

Lung cancer remains one of the deadliest cancers in the world, and 85% of them are non-small cell lung cancer (NSCLC)^1^. According to statistics, lung adenocarcinoma (LUAD) is a major subtype of NSCLC, accounting for approximately 40% of all patients^2^. Despite significant advances in the treatment of LUAD, the 5-year overall survival (OS) rate for LUAD is less than 20%^3^.

In recent years, with the rapid development of immunotherapy, especially the application of immune checkpoint inhibitors (ICIs), the prognosis of lung cancer patients has been improved significantly^4,5^. However, not all patients respond to ICIs, and a minority of patients do not benefit from ICIs^6^. In addition, it is worth noting that similar to chemotherapy or targeted therapy, a majority of patients who initially respond to ICI therapy and then develop primary or secondary resistance.

A growing number of studies suggest that tumor microenvironment (TME) plays an important role in cancer development and antitumor processes and may involve in immunotherapy resistance^7^. TME contains various immune cells, stromal cells and extracellular matrix molecules, of which tumor-infiltrating lymphocytes (TILs) are the main immune cells exerting anti-tumor activity^8,9^. TILs have been reported as predictive immune efficacy biomarkers for lung cancer^10^. However, TILs, especially CD8 + T cells, eventually developed a state of dysfunction known as T-cell exhaustion during long-term tumor fight^11^. The exhausted T cells have been demonstrated to exhibit overexpression of inhibitory receptors, metabolic dysregulation, epigenetic reprogramming and loss of effector functions^12–14^. And studies have proven that exhausted T cells can induce immune tolerance^15^. Immunotherapy depends on the activation of T cells to eliminate tumors. However, the emergence of T-cell exhaustion impedes it capacity of anti-tumor and facilitates immune escape. The formation of exhausted T cells is regulated by multiple factors, and a deep understanding of key genes that regulated T cell exhaustion might help to interpret the underlying mechanism.

Therefore, our study aimed to explored the crucial molecules of T cell exhaustion in LUAD, and attempted to construct a prognostic signature and nomogram model to predict prognosis and immunotherapy response for LUAD patients.

## Methods

### Publicly available datasets

We downloaded the normalized RNA sequencing data (transcripts per million, TPM) and raw counts data of 539 LUAD patients from the cancer genome atlas (TCGA) (https://portal.gdc.cancer.gov/) database. The normalized matrix files of GSE72094 cohort from the Gene Expression Omnibus (GEO) database (https://www.ncbi.nlm.nih.gov/), which had included 442 LUAD patients, used as an independent external cohort for risk model validation. The patients’ characteristics and other information of TCGA-LUAD and GSE72094 cohorts were shown in Supplementary file 1: Table S1. In addition, immunotherapy cohorts including GSE135222 and GSE91061 from GEO database were included for validation of immunotherapy response. Probe IDs were converted to gene symbols according to the platform annotation file. If multiple probes matched the same gene symbol, their median expression values were selected. The information of TCGA-LUAD and GEO datasets was summarized in Supplementary file 1: Table S2.

### Weighted correction network analysis (WGCNA)

Weighted correction network analysis (WGCNA) was used to construct co-expression networks of scale-free distributions of genes based on correlations to gene expression, from which the most relevant modules to clinical features were identified^16^. In our study, we used the "pickSoftThreshold" method to choose an appropriate soft threshold parameter. Then, the adjacency was transformed into a topological overlap matrix (TOM) and genes with similar expression patterns were divided into the same module. Finally, genes from modules that had high correlation coefficients with T-cell exhaustion and dysfunction were identified for subsequent analysis.

### Consensus clustering analysis

The “ConsensusClusterPlus” R package was used to perform the unsupervised consensus clustering analysis^17^. We used 80% of the items for subsampling and divided each subsample into groups by the k-means algorithm, repeating this clustering process 1000 times. Then, The cumulative distribution function (CDF) curve and consensus matrix are used to identify the optimal k-value. Based on the optimal k-value, patients were clustered into sub-clusters. In addition, the principal component analysis (PCA) and principal coordinate analysis (PCoA) were performed to show the distribution difference of sub-clusters.

### Functional enrichment analysis

Enrichment analysis in Gene Ontology (GO) and the Kyoto Encyclopedia of Genes and Genomes (KEGG) were based on the “Clusterprofiler” R package, where “adjusted *p* < 0.05” was considered significant^18^. The gene sets of “hallmark” from the Molecular Signatures Database (MSigDB) were analyzed through the gene set enrichment analysis software (v4.3.2). *P*.adjust < 0.05 and q values < 0.25 were considered statistically significant^19,20^.

### Tumor immune microenvironment evaluation

The ESTIMATE algorithm tool was used to was used to calculate stromal, immune and estimated scores^21^. The CIBERSORTx (https://cibersort.stanford.edu/) was used to evaluate the composition of 22 kinds of tumor-infiltrating immune cells based on the principle of linear support vector regression^22^. Single sample gene set enrichment analysis (ssGSEA) based on the R package GSVA was used to quantify single sample immune cell infiltration scores. The immune cell abundance identifier (ImmuCellAI) (http://bioinfo.life.hust.edu.cn/ImmuCellAI) was utilized to obtain the scores of T cell exhaustion^23^. Moreover, we compared the mRNA expression levels of immune checkpoint suppressor molecules including programed cell death protein 1 (PD1), programmed death ligand 1 (PD-L1), cytotoxic T-lymphocyte associated protein 4 (CTLA4), lymphocyte activating 3 (LAG3), T cell immunoreceptor with Ig and ITIM domains (TIGIT), hepatitis A virus cellular receptor 2 (HAVCR2) and other immunosuppressive molecule in two clusters.

### Development and verification of prognostic signature

Firstly, differential genes between cluster 1 and cluster 2 in TCGA cohort were analyzed with the “DESeq2” R package based on the mRNA expression of raw counts data^24^. The threshold of adjusted *P* value less than 0.05 (*P* < 0.05) and the absolute log2 fold-change greater than 1 (|log2FC|> 1) were define differential genes. Next, univariate cox regression analysis was utilized to screen differential genes that were associated with OS in TCGA and GSE72094 cohort using the “survival” R package (*P* < 0.05). Then, we performed machine learning algorithms including the least absolute shrinkage and selection operator (LASSO) regression analysis^25^ and random forest (RF) algorithm^26^ to improve the accuracy and reliability of prognostic signature using the “glmnet” and “randomForestSRC” R packages, respectively. Furthermore, we used the intersection of LASSO and the RF to determine candidate genes, followed by multivariate cox regression analysis. According to the results of the multivariate cox analysis, the prognostic signature was established as follows: Riskscore = $\sum_{i=1}^{n}\left(Coef\times Ni\right)$, where Coef referred the coefficient of gene i and Ni represented the expression value of gene i.

To further validate the predictive power of the prognostic signature, we performed Kaplan–Meier survival analysis in the TCGA-LUAD and GSE72094 cohorts, where the cutoff value was set to the median riskscore. Time-dependent receiver operating characteristic curve (ROC) were plotted using the “timeROC” R package to predict OS at 1, 2 and 3 years in the training dataset (TCGA-LUAD) and validation cohort (GSE72094). Finally, we further analyzed the relationship between the prognostic signature and clinical characteristics (including age, gender and stage) to clarify the independent prognostic value of riskscore using multivariate Cox regression analyses.

### Development and evaluation of the nomogram

We performed the univariate and multivariate Cox regression analyses for clinical parameters and riskscore. In the multivariate Cox model, variables with *P* < 0.05 were included in the construction of the nomogram. The calibration analysis and time-dependent ROC curves were used to assess the prognostic accuracy of the nomogram model. Decision curve analysis (DCA) curves used to evaluate the net benefit of the nomogram.

### Immunotherapeutic response prediction

Tumor Immune Dysfunction and Exclusion (TIDE) was a computational method to predict ICIs response^27^. Based on transcriptomic data from TCGA cohort, T-cell dysfunction scores and TIDE scores were calculated through an online website (http://tide.dfci.harvard.edu). The Cancer Immunome Atlas (TCIA) database describes the immune landscape of 20 solid tumors and developed a novel score called “Immunophenscore” (IPS), which also predicted the response to ICIs. We calculated each patients’ IPS scores through an online website (https://tcia.at/)^28^. Tumor mutational load (TMB) was a valuable biomarker to assess the efficiency of immunotherapy^29^. We utilized the “TCGAbiolinks” R package to download somatic mutation data, and then calculated TMB values for each LUAD patient in the TCGA cohort based on the “Maftools” R package^30^. In addition, the mRNA expression of PD-1, PD-L1 and CTLA4 was analyzed in low or high-risk groups.

### Chemotherapeutic drug sensitivity prediction

We utilized the “pRophetic” R package to estimate the half-maximal inhibitory concentrations (IC50) of drugs to predict the sensitivity of LUAD patients to chemotherapy and targeted therapies^31^.

### Statistical analysis

All statistical analyses were performed using R software (version 4.2.2). Wilcoxon test was used to compare the differences between groups. The log-rank test was used to compare Kaplan–Meier survival curves. Univariate and multivariate Cox analyses were performed to establish independent prognostic factors. All *P* values were two-sided and less than 0.05 were considered statistically significant.

## Results

### Identification of Tex-related genes and functional analysis

**Figure 1:**
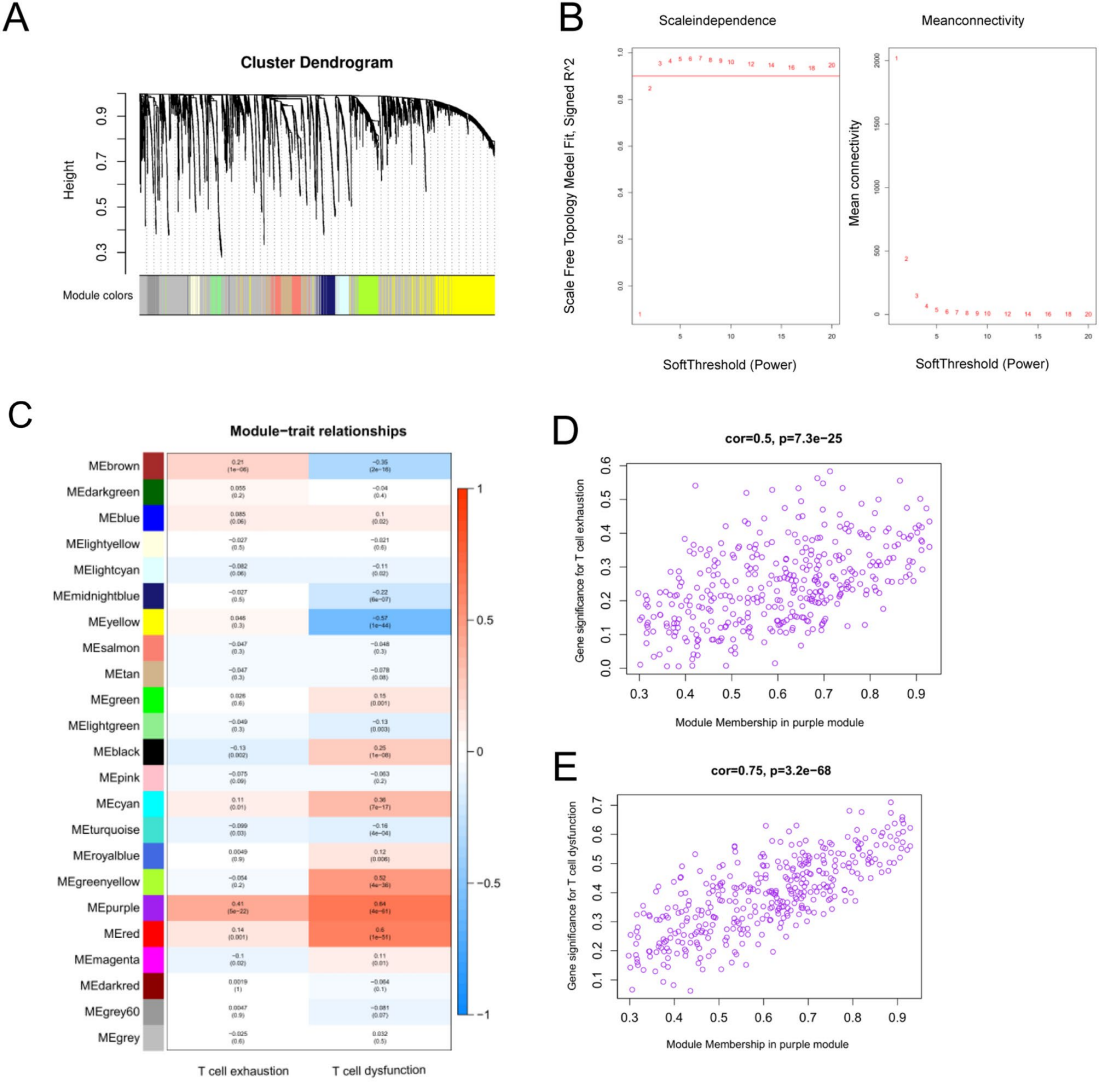
WGCNA analysis in TCGA cohort. (**A**) The coexpression network. (**B**) The soft threshold power of WGCNA. The left one showed the analysis of the scale-free index for various soft threshold powers. The right one showed the analysis of the average connectivity for various soft threshold powers. (**C**) Heatmap displayed correlation between module eigengenes and T-cell exhaustion and T-cell dysfunction. (**D**) Identification of the modules most significantly associated with T-cell exhaustion. (**E**) Identification of modules most significantly associated with T-cell dysfunction.

WGCNA analysis was conducted according to the expression of LUAD mRNA in the TCGA cohort (Fig. 1A). We choose a soft threshold of 4, which met the scale-free network rule (Fig. 1B). Figure 1C–E showed that the purple module was the most significantly related to T-cell exhaustion (Tex) (cor = 0.5, *P* = 7.3e-25) and T cell dysfunction (cor = 0.75, *P* = 3.2e-68). Therefore, the purple module (n = 371) was characterized as Tex-related genes. GO and KEGG enrichment analysis revealed possible biological processes involved in Tex-related genes (Supplementary file 2: Fig. S1).

### Identification of Tex subgroups

**Figure 2:**
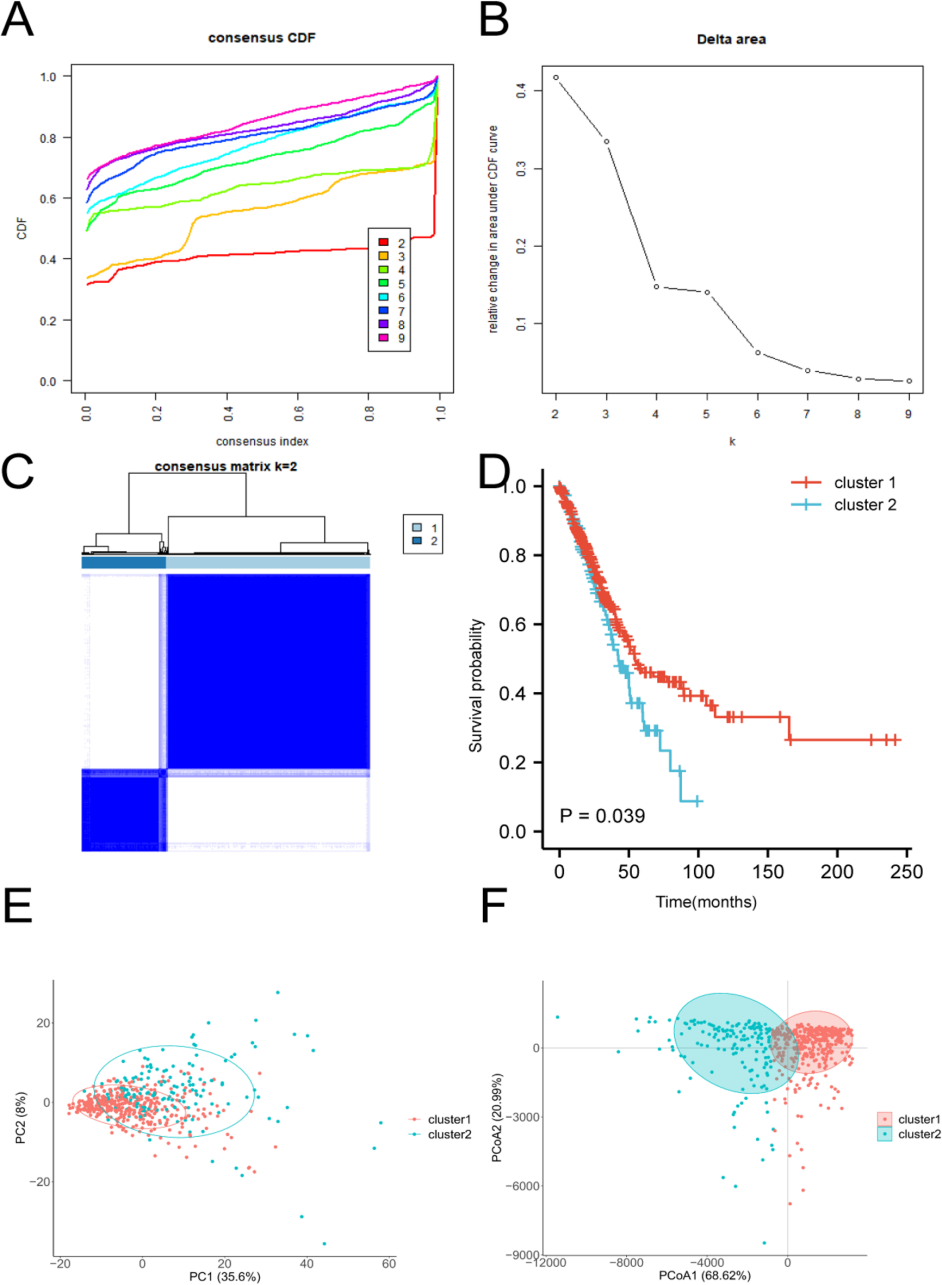
Unsupervised consensus cluster analysis for LUAD patients in the TCGA cohort based on T-cell exhaustion related genes. (**A**) Consensus CDF from k = 2–9. (**B**) Delta area under the cumulative distribution function (CDF) curve of different clusters ranging from k = 2–9. (**C**) Consensus matrix for k = 2. (**D**) The overall survival (OS) probability of the patients in the two clusters. (**E**) The principal component analysis (PCA) and principal coordinate analysis (PCoA) of two clusters.

Tex subgroups were identified by using an unsupervised clustering approach based on the expression levels of Tex related genes in TCGA. Based on the consensus CDF curve (Fig. 2A), the relative change in area under the CDF curve (Fig. 2B), and the consensus matrix (Fig. 2C), we finally selected k = 2 as the best cluster. Two different subgroups were identified, including 375 patients in cluster 1 and 155 patients in cluster 2. It is clear that patients in cluster 1 had a better OS than those in cluster 2 (*P* = 0.039, Fig. 2D). Two significantly different subgroups were further confirmed by the results of PCA as well as PCoA analysis (Fig. 2E–F). In addition, we compared the differences in clinical characteristics between cluster 1 and cluster 2. The results in Table 1 showed that patients in cluster 2 had a higher proportion of female and older patients (> 65 years), and there was no difference in the tumor stage between two subgroups.

**Table 1:** The clinical characteristics between cluster 1 and cluster 2.

Characteristic	Cluster 1	Cluster 2	*P* value
Age			0.025
≤ 65	195 (52.8%)	62 (41.1%)	
> 65	174 (47.2%)	89 (58.9%)	
Unknown	11 (2.9%)	8 (5%)	
Gender			0.004
Female	188 (49.5%)	101 (63.5%)	
Male	192 (50.5%)	58 (36.5%)	
T stage			0.276
T1	116 (30.6%)	60 (38.2%)	
T2	214 (56.5%)	78 (49.7%)	
T3	36 (9.5%)	13 (8.3%)	
T4	13 (3.4%)	6 (3.8%)	
Unknown	1 (0.3%)	2 (1.3%)	
N stage			0.047
N0	247 (67.3%)	103 (66%)	
N1	59 (16.1%)	38 (24.4%)	
N2	60 (16.3%)	14 (9%)	
N3	1 (0.3%)	1 (0.6%)	
Unknown	13 (3.4%)	3 (1.9%)	
M stage			0.431
M0	251 (93%)	114 (95%)	
M1	19 (7%)	6 (5%)	
Unknown	110 (28.9%)	39 (24.5%)	
Pathological stage			0.426
I	209 (55.9%)	87 (55.4%)	
II	81 (21.7%)	44 (28%)	
III	64 (17.1%)	20 (12.7%)	
IV	20 (5.3%)	6 (3.8%)	
Unknown	6 (1.6%)	2 (1.3%)	

### Immunological landscape of Tex subtypes

**Figure 3:**
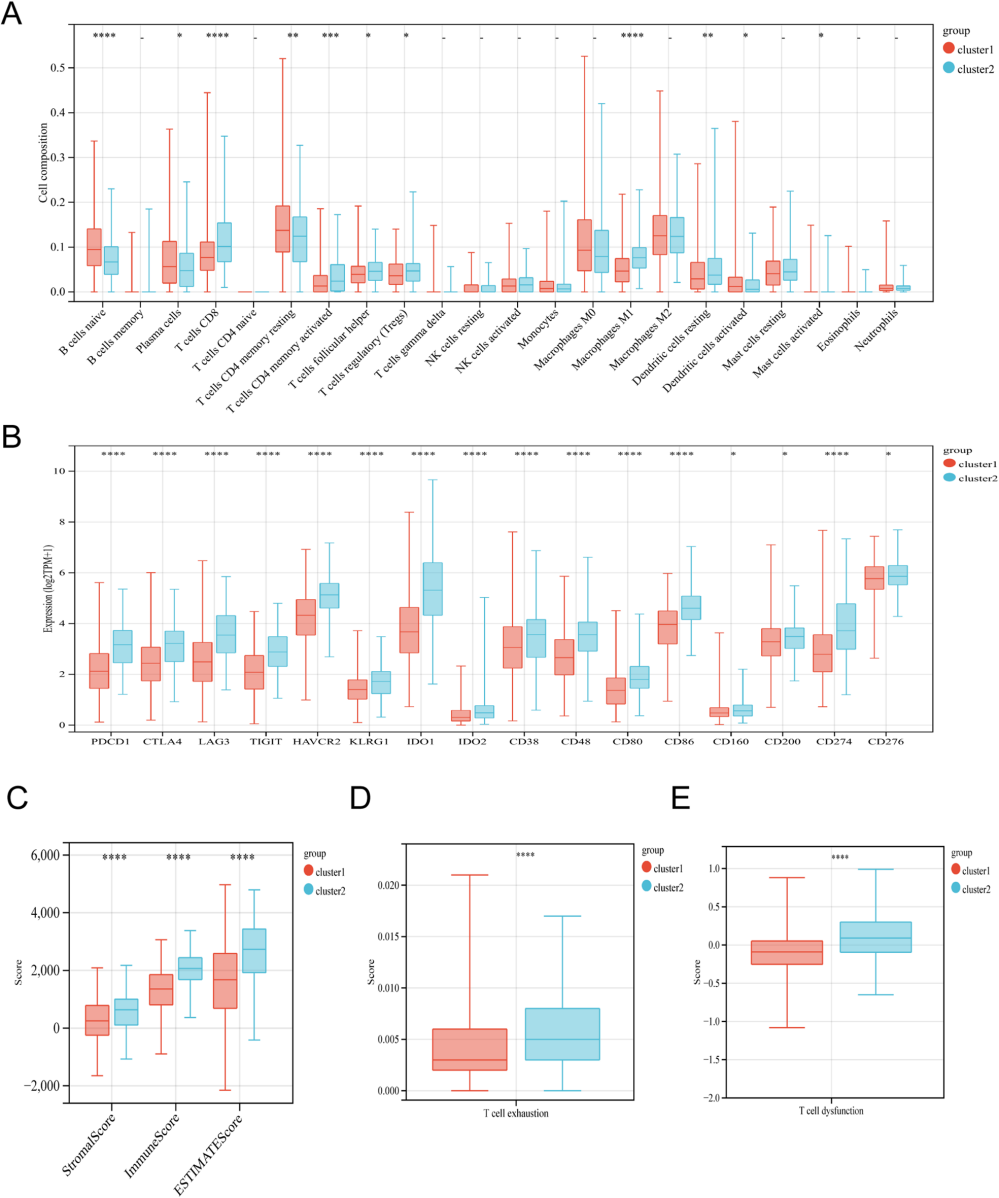
Immune analysis of different clusters. (**A**) Differences in abundance of tumor-infiltrating immune cells in different clusters based on CIBERSORT. (**B**) Differential mRNA expression of immune checkpoints in different clusters. (**C**) The stromal, immune and estimate scores between two clusters. (**D**) Differences in T-cell exhasution score between different clusters. (**E**) Differences in T-cell dysfunction score between different clusters. (*p < 0.05; **p < 0.01; ***p < 0.001; ****p < 0.0001).

**Figure 4:**
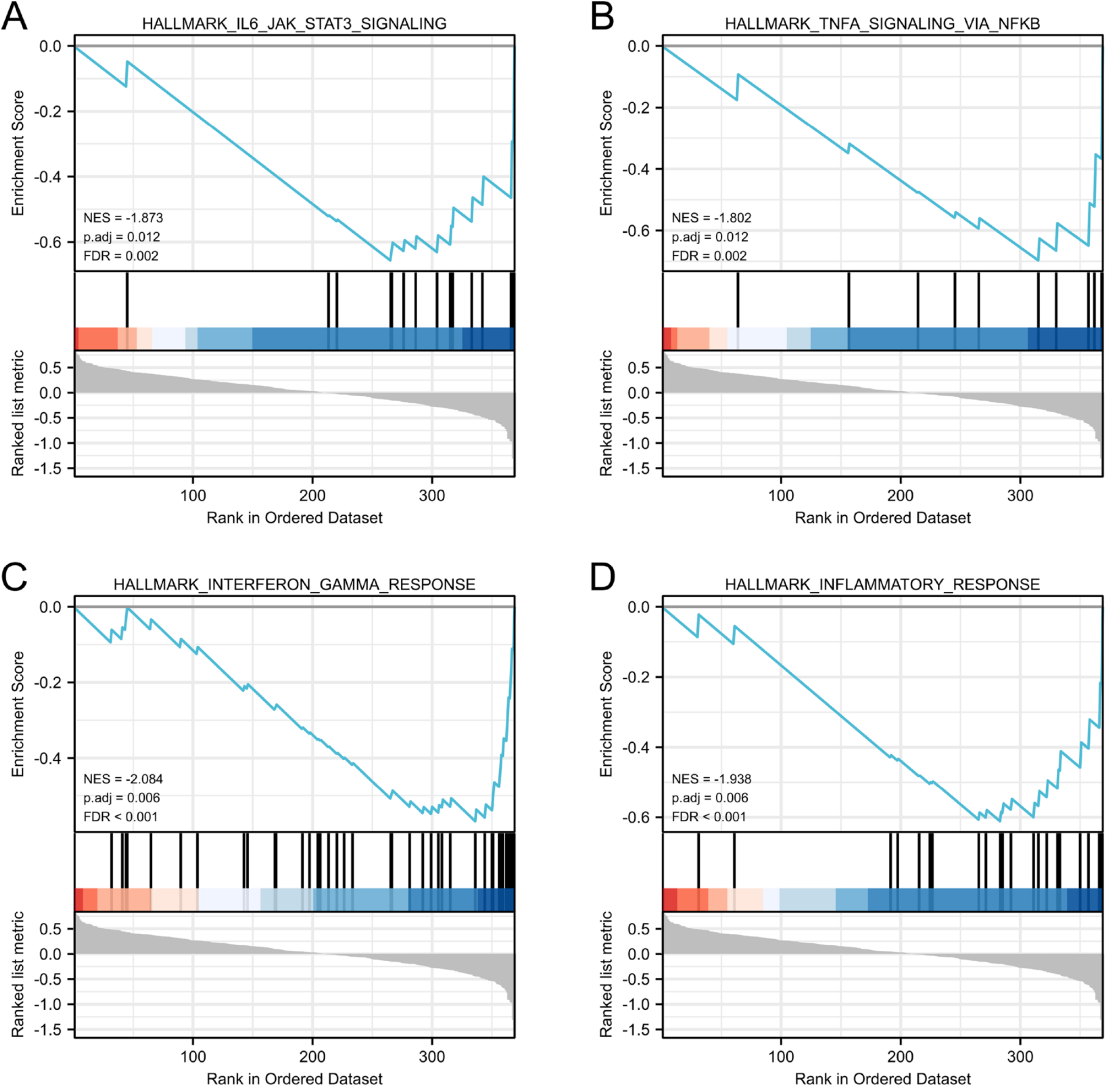
Gene set enrichment analysis (GSEA) showed revealed enrichment for biological processes associated with immunosuppression.

First, we calculated the composition of immune cells between clsuter 1 and cluster 2 by the CIBERSORT algorithm (Fig. 3A). Comparing with cluster 1, cluster 2 had significantly more abundance of CD8 T cell and CD4 memory activated T cell. However, despite having higher CD4/CD8 T cell levels, patients in cluster 2 had worse prognosis than those in cluster 1. This was contradictory to the fact that high levels of tumor infiltrating CD4/CD8 T cells were generally associated with better prognosis. Therefore, we hypothesized that these CD4/CD8 T cells in cluster 2 were prone to exhaustion. Next, we compared the mRNA expression levels of immune checkpoint suppressor molecules in the two clusters. The results showed that PDCD1 (PD1), CD274 (PD-L1), CTLA4, LAG3, TIGIT, HAVCR2 and other immunosuppressive molecules were upregulated in cluster 2 (Fig. 3B). Then, we assessed the TME score using an estimation algorithm. The results displayed that cluster 2 had higher immune, stromal, and estimate scores than those in cluster 1 (Fig. 3C). In addition, we also found that T-cells exhaustion as well as T-cell dysfunction scores were significantly higher in cluster 2 than in cluster 1 (Fig. 3D–E). Finally, we performed GSEA analysis based on the Tex-related genes identified by WGCNA, and the results showed that cluster 2 was enriched in IL6/JAK/STAT3 signaling, interferon_gamma response, TNFA signaling via NFKB and inflammatory response (Fig. 4). Based on the above results, we inferred that cluster 2 mediated the suppressive immune microenvironment associated with T-cell exhaustion.

### Machine learning identifies hub genes and functional analysis

**Figure 5:**
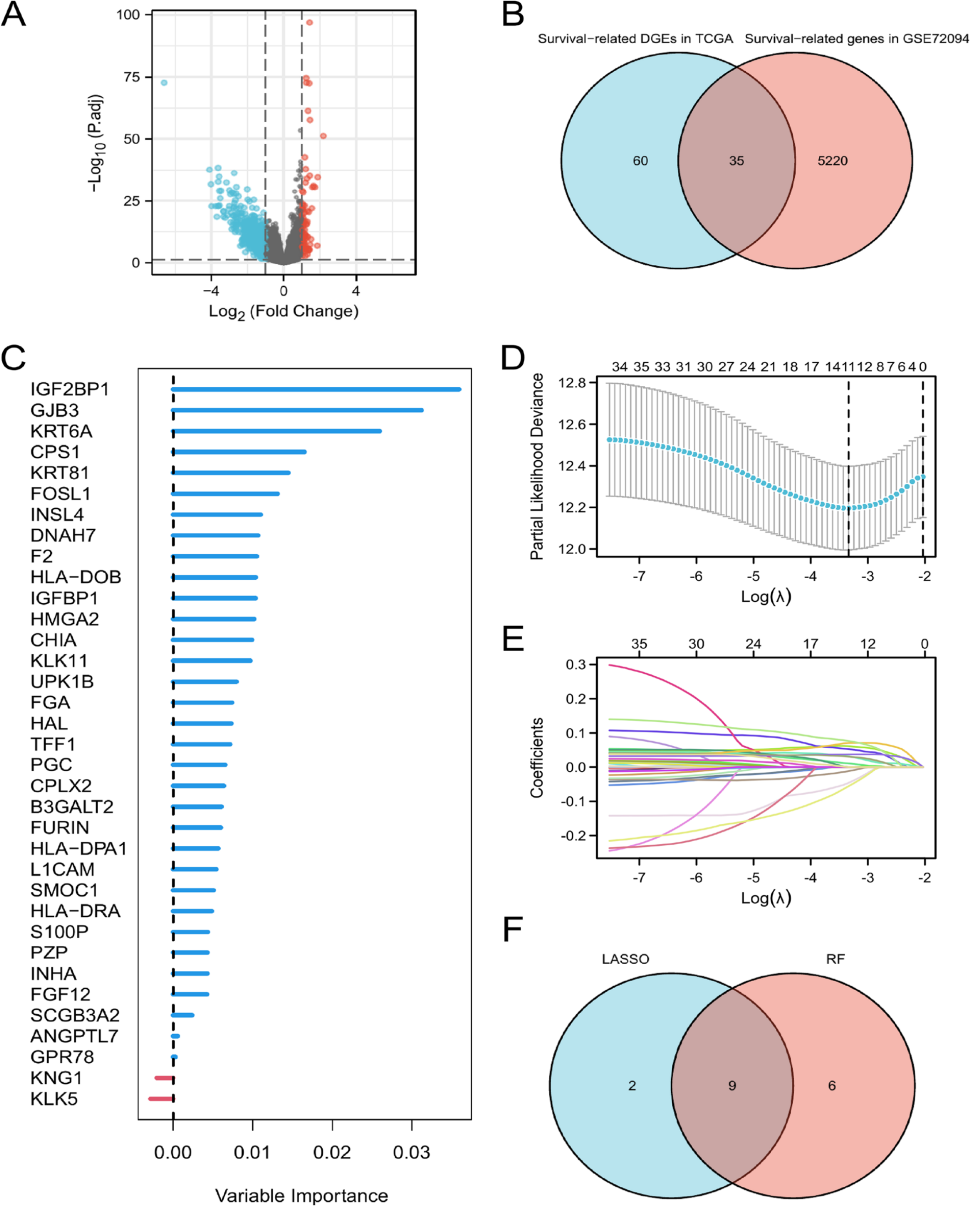
Machine learning identifies hub genes associated with involved in the regulation of T-cell exhaustion. (**A**) Volcano map shows differential genes between cluster1 and cluster 2 in the TCGA. (**B**) 35 intersected differential genes associated with OS in TCGA and GSE72094 cohort. (**C**) The importance of 35 genes using random forest (RF). (**D**–**E**) 11 hub genes using the least absolute shrinkage and selection operator (LASSO) regression analysis. (**F**) 9 intersected hub genes based on 11 genes in LASSO and 15 top genes in RF.

First of all, we performed differential analysis of mRNA expression in cluster1 and cluster 2 based on the TCGA cohort to identify differential genes (Fig. 5A). Then, univariate cox regression analysis was utilized to screen differential genes that were associated with OS in TCGA and GSE72094 cohort. Venn diagram showed a total of 35 genes were identified as Tex related prognostic differential genes (Fig. 5B). Next, top 15 genes were selected using RF (Fig. 5C) and 11 genes were selected using LASSO (Fig. 5D–E). Finally, 9 intersected genes (including CPS1, FOSL1, GJB3, HLA-DOB, IGF2BP1, IGFBP1, KLK11, KRT6A, KRT81) were identified as hub genes (Fig. 5F). GO and KEGG analysis showed that these nine hub genes were involved in response to cAMP, carbon–nitrogen ligase activity, MHC class II receptor activity, gap junction channel activity and so on (all adjusted *P* < 0.05, Supplementary file 2: Fig. S2).

### Construction of prognostic signature

**Figure 6:**
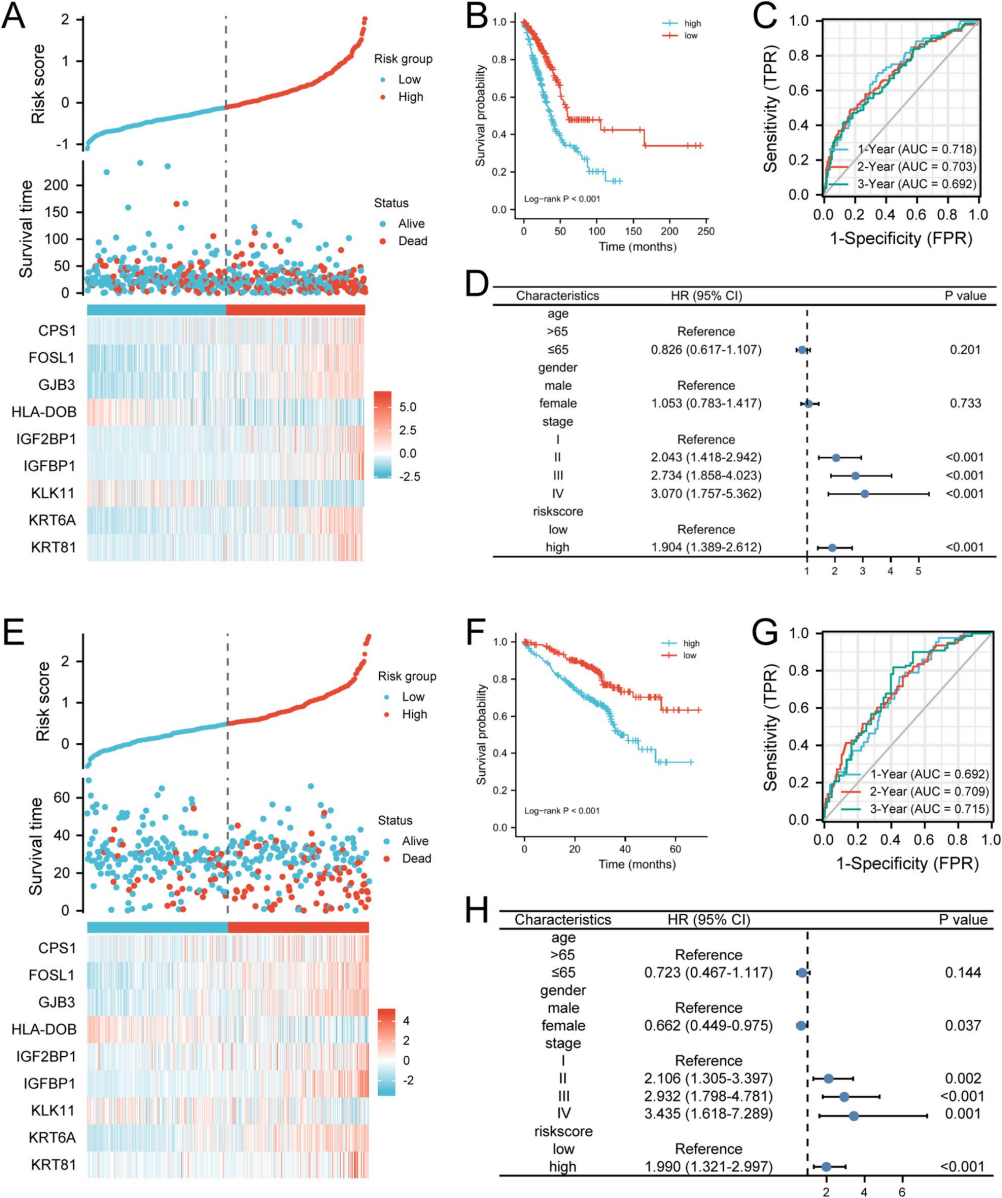
The establishment and validation of the riskscore in TCGA and GSE72094 cohort respectively. (**A**, **E**) Risk map for prognostic signature and heat map for hub genes expression. (**B**, **F**) Kaplan–Meier curves for the riskscore. (**C**, **G**) The prognostic signature predict time-dependent ROC curves at 1, 2, and 3 years OS. (**D**, **H**) Multivariate cox regression analysis to verify the independent predictive value of the riskscore.

The hub genes were enrolled in multivariate cox analysis to construct the prognostic signature: riskscore = (CPS1 × 0.0376) + (FOSL1 × 0.0731) + (GJB3 × 0.0641)—(HLA-DOB × 0.1590) + (IGF2BP1 × 0.0732) + (IGFBP1 × 0.1072)—(KLK11 × 0.0445) + (KRT6A × 0.0460) + (KRT81 × 0.0439)—0.2897. Patients in the TCGA cohort were classified as low- or high-risk group when the cutoff value was defined as the median riskscore (Fig. 6A). The Kaplan–Meier survival curves showed showed that the median OS of the low-risk group was longer than that of the low-risk group (*P* < 0.001, Fig. 6B). The time-dependent ROC curve displayed that the risk score had a good predictive performance, with an area under the curve (AUC) of 0.718, 0.703, 0.692 at 1, 2, and 3 years, respectively (Fig. 6C). In addition, when the riskscore was included in multivariate cox analysis combined with age, gender, and stage, it still was demonstrated as an independent prognostic factor for OS (HR = 1.904; 95% CI 1.389–2.612; *P* < 0.001; Fig. 6D). To further confirmed the reliability of the riskscore, we used the same formula to calculate the riskscore for patients in the GSE72094 cohort and used it for external validation. Similar to the previous results (Fig. 6E–H), the riskscore was an independent prognostic factor (HR = 1.990; 95% CI: 1.321–2.997; *P* < 0.001) as well and had good predictive power with an AUC at 1-, 2-, 3-year of 0.692, 0.709, 0.715. Moreover, we further stratified Tex subgroups based on riskscore and found that cluster 2 with high riskscore had the worst prognosis (Supplementary file 2: Fig. S3).

### Correlation between the riskscore and clinical features

**Figure 7:**
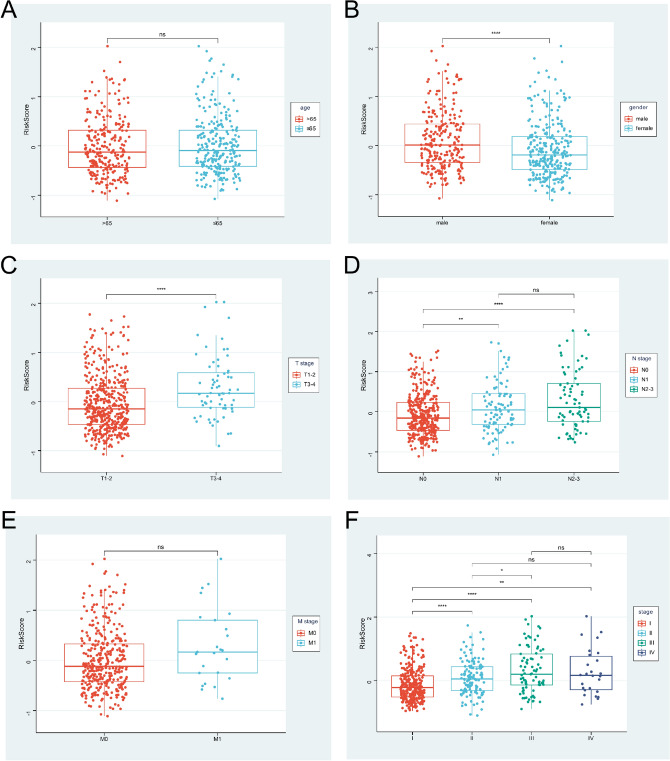
Correlation between the riskscore and clinical features. (**A**) age, (**B**) gender, (**C**) T stage, (**D**) N stage, (**E**) M stage, (**F**) pathological stage. (ns *p* > 0.05; *p < 0.05; **p < 0.01; ***p < 0.001; ****p < 0.0001).

We explored relationships between the riskscore and clinical features in TCGA cohort and found that gender and TNM stage were significant correlated with the riskscore. Male, T3-4 stage, more lymph node metastases, distant metastases, and advanced stage patients tended to have higher riskscores (Fig. 7).

### Construction and evaluation of nomogram model

**Figure 8:**
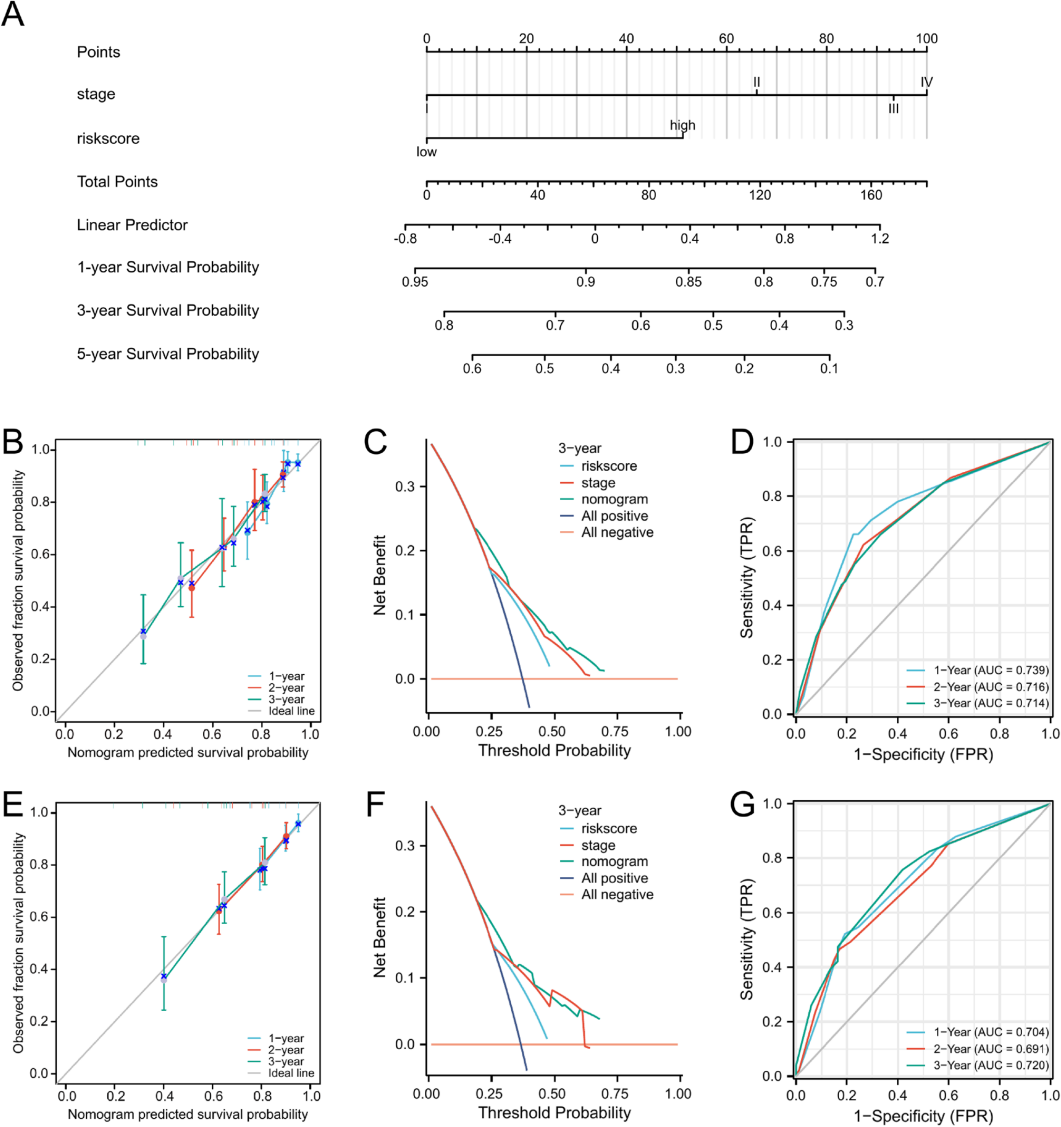
Construction and evaluation of the nomogram. (**A**) The nomogram combined the riskscore with stage for prognostic prediction of a patient with LUAD in the TCGA cohort. (**B**, **E**) Calibration curves of 1-year, 2-year, and 3-year OS for LUAD patients in the TCGA cohort and GSE72094 cohort. (**C**, **F**) Decision curve analysis of 3-year survival benefit in the TCGA cohort and GSE72094 cohort. (**D**, **G**) Time-dependent receiver operating characteristic (ROC) curves of the nomogram to predict 1-year, 2-year, and 3-year OS in the TCGA cohort and GSE72094 cohort.

The above results suggested that both tumor stage and risk score were independent prognostic factors. Thus, a nomogram was constructed to predict survival combined pathological stage with the riskscore (Fig. 8A). Calibration curves showed substantial agreement between the expected and actual probabilities of nomgram in predicting 1, 2, and 3-year survival (Fig. 8B). DCA curves showed the nomogram model had a greater net benefit in predicting the 3‐year OS (Fig. 8C). The time-dependent ROC curves showed a higher accuracy of the nomogram in predicting survival, with AUC values of 0.739, 0.716, 0.714 at 1, 2, and 3 years OS (Fig. 8D). Meanwhile, the model was validated using the GSE72094 cohort, and the results were similar to the results in TCGA cohort (Fig. 8E–G).

### Immunotherapeutic response prediction

**Figure 9:**
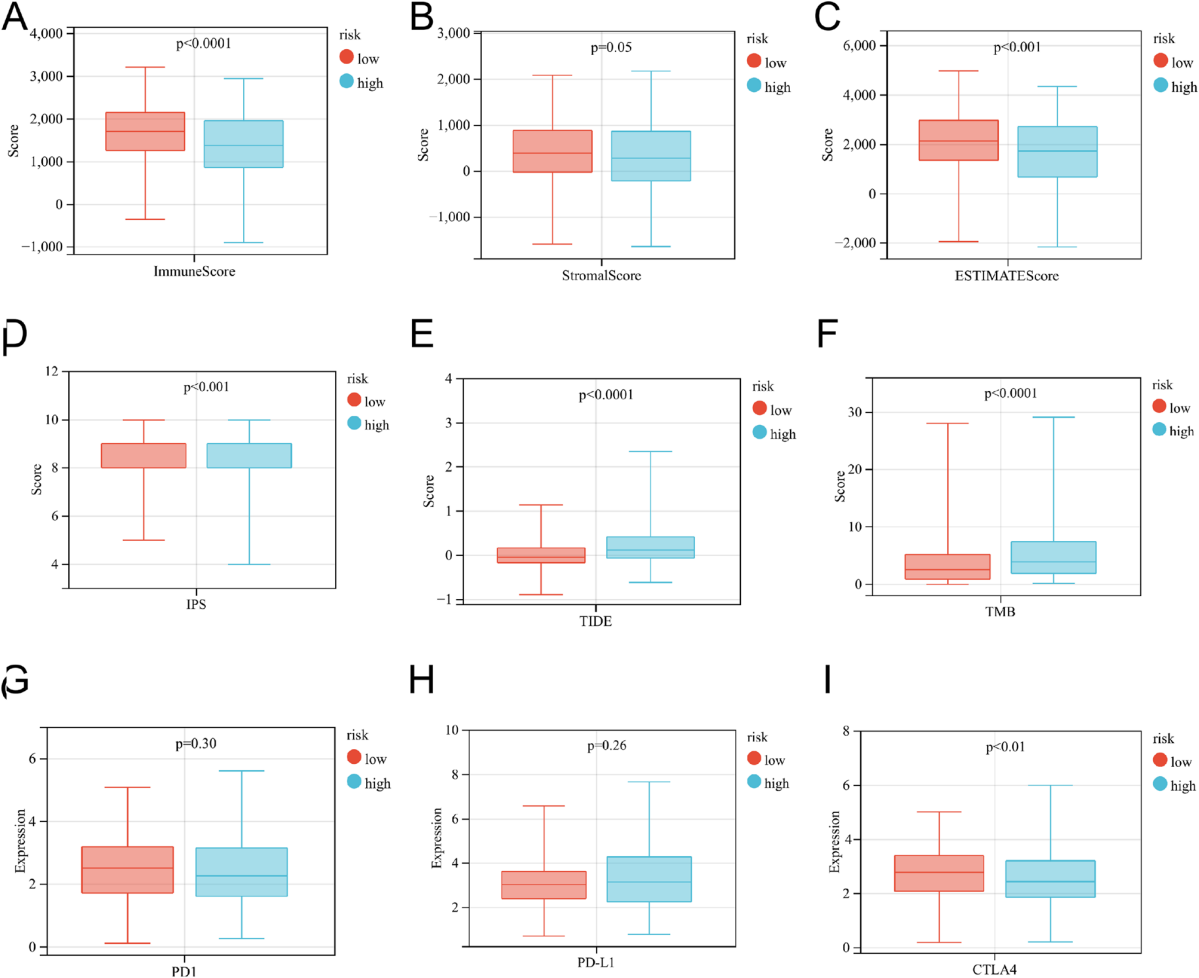
The distribution of immune score (**A**), stromal score (**B**), estimate score (**C**), IPS score (**D**), TIDE score (**E**), TMB (**F**), and the mRNA expression of immune checkpoint inhibitors (**G**–**I**) in different risk groups.

To assess ability of the riskscore as a biomarker for predicting immunotherapeutic response, we estimated the distribution of TME scores, IPS score, TIDE scores, TMB, immune cell infiltration scores and the mRNA expression of HLA and immune checkpoint inhibitors in different risk groups. The results of the TME score analysis indicated that the low-risk group had higher immune score (*P* < 0.001; Fig. 9A), stromal score (*P* = 0.05; Fig. 9B), and estimate score (*P* < 0.01; Fig. 9C). Further, TCIA database analysis revealed the low-risk group had higher IPS (*P* < 0.001; Fig. 9D). We also found that TIDE scores and TMB were higher in the high-risk group (both *P* < 0.001; Fig. 9E–F). In addition, our study results showed that the mRNA expression of PD-1 and PD-L1 were not significantly different between the two groups, except for CTLA4, which was significantly upregulated in the low-risk group. (Fig. 9G–I). We also observed that the low-risk group had significantly higher immune cell infiltration than the high-risk group, including activated CD8T cells, cytotoxic cells, dendritic cells, T helper cells, and so on (Supplementary file 2: Fig. S4). Lastly, we investigated the relationship between high- and low-risk groups and human leukocyte antigens (HLA). Except for HLA-A/B/C/F/G, the expression of HLA appeared to be higher in low-risk group (Supplementary file 2: Fig. S5).

**Figure 10:**
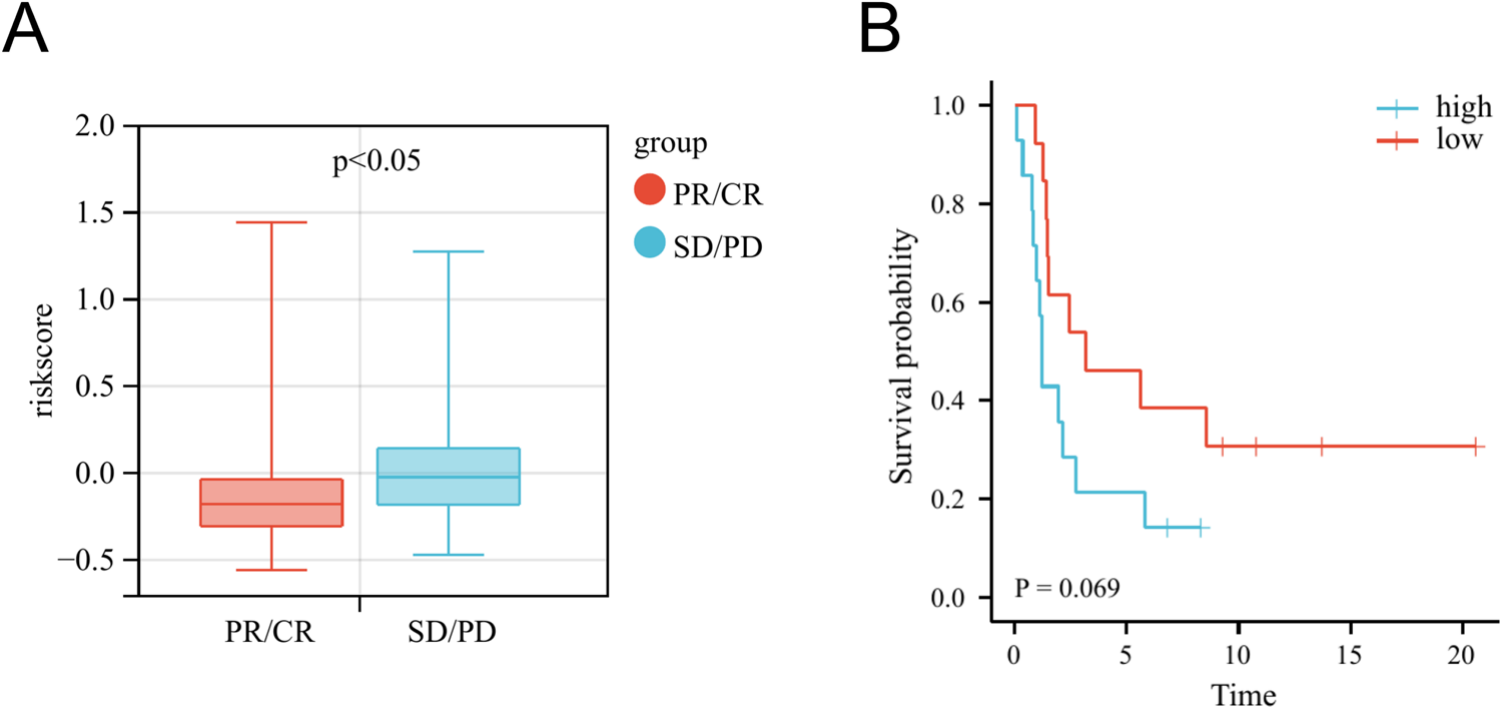
(**A**) Differences in immunotherapy response between low- and high-risk groups in the GSE91061 dataset. (**B**) Progression-free survival for patient with anti-PD-1/PD-L1 therapy between the low- and high-risk groups in the GSE135222 dataset.

To further validate that the riskscore could effectively predict immune efficacy response, we also included two immunotherapy cohorts to evaluate the riskscore model. GSE91061, which including 109 melanoma samples with anti-CTLA4 and anti-PD1 therapy demonstrated patients with complete response (CR) or partial response (PR) had a lower riskscore than those with stable disease (SD) or progressive disease (PD) (Fig. 10A). Moreover, GSE135222, which including 27 advanced NSCLC samples with anti-PD-1/PD-L1 confirmed that the low-risk group had better PFS compared to the high-risk group (Fig. 10B).

### Chemotherapeutic drug sensitivity prediction

**Figure 11:**
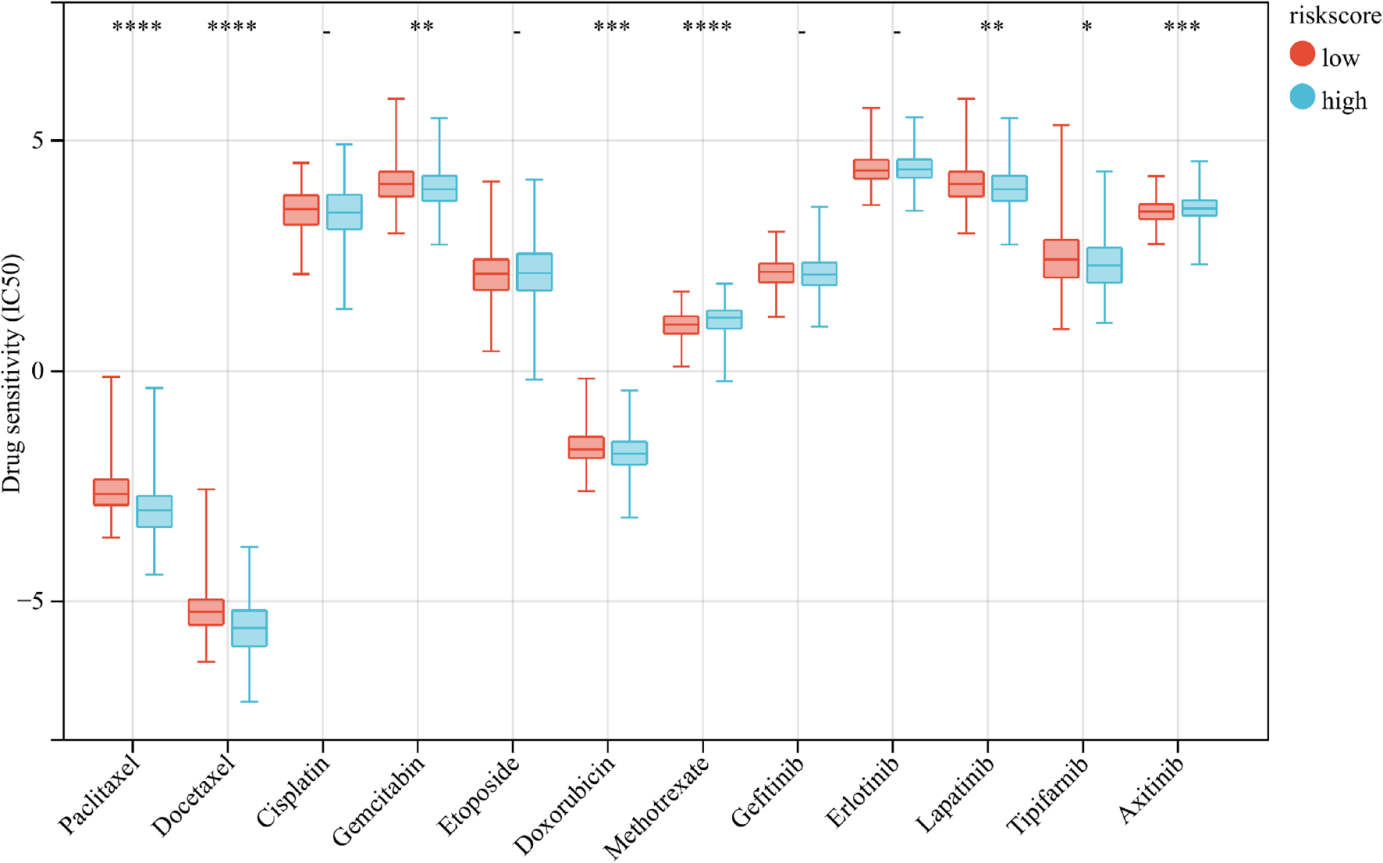
The IC50 values of chemotherapy and targeted drugs for LUAD in different risk groups. (*p < 0.05; **p < 0.01; ***p < 0.001; ****p < 0.0001).

The IC50 values of common chemotherapy and targeted drugs for LUAD were calculated to further explored the drug sensitivity between low- and high-risk groups. By comparing the difference in IC50 values in two risk groups (Fig. 11), we found that patients in the low-risk group had a higher sensitivity to paclitaxel, docetaxel, doxorubicin, gefitinib, lapatinib, and tipifarnib while patients in high-risk group had a higher sensitivity to axitinib and methotrexate. Besides, patients in the high and low risk groups showed no significant difference in the sensitivity of cisplatin, etoposide, erlotinib and gefitinib.

## Discussion

T-cell exhaustion is considered to be one of the most crucial reasons for immune resistance^32^. However, it is still not known what regulates T-cell exhaustion in the tumor microenvironment. Therefore, an in-depth exploration of key regulatory genes of T-cell exhaustion could help to understand the potential mechanisms of immune resistance and accordingly provide a theoretical basis for clinical decision making.

In the present study, we used WGCNA to identify the module of genes most related to T-cell exhaustion and T-cell dysfunction. Next, unsupervised clustering approach based on the expression levels of these genes divided patients into two different subgroups. Obviously, patients in Cluster 1 had better OS compared with patients in cluster 2. It was intriguing that patients in cluster 2 had higher abundance of CD8 T cell and CD4 memory activated T cell compared to group 1. This is contradictory to the fact that high levels of tumor-infiltrating CD4/CD8 T cells usually associated with good outcome^33,34^. Therefore, we hypothesized that these CD4/CD8 T cells in cluster 2 maybe exhausted T cells. Research had revealed that exhausted T cells often exhibit persistent high expression of multiple suppressive receptors, such as PD-1, CTLA-4, and LAG-3^13^. In our study, we found that PD-1, CTLA4, LAG3, TIGIT, HAVCR2 and other immunosuppressive molecules were upregulated in cluster 2. In addition, GSEA analysis cluster 2 was enriched in IL6/JAK/STAT3 signaling, interferon_gamma response, TNF_alpha signaling via NFKB and inflammatory response. Studies had demonstrated that JAK/STAT signaling pathway^35,36^ the IFN-γsignaling pathway^37^ and TNF-ɑ signaling via NFKB^38^ caused immune escape by upregulating PD-L1 expression. Therefore, we inferred that cluster 2 might mediate the suppressive immune microenvironment.

Next, we further analyzed the significantly differentially expressed genes between the two groups and used machine learning algorithms to finally identify nine key regulatory genes, including CPS1, FOSL1, GJB3, IGF2BP1, HLA-DOB, IGFBP1, KRT6A, KLK11, KRT81, that may be involved in regulating T cell exhaustion. The enzyme carbamoyl phosphate synthetase 1 (CPS1) is a key rate-limiting enzyme in the urea cycle, involving in ammonium conversion and mediated arginine metabolism and pyrimidine metabolism^39^. T cell is highly sensitive to extracellular levels of arginine^40^, and arginine is essential for T cell function^41,42^. It has been shown that low arginine mediates an immunosuppressive microenvironment, which may suppress T-cell responses by providing a brake on T-cell proliferation^43^. In addition, CPS1 deficiency could lead to hyperammonemia, which impair mitochondrial function, reduce ATP synthesis, and increase free radical formation, leading to oxidative stress^44^, thereby possibly inducing T-cell exhaustion. FOSL1 encodes Fra-1, which is initially found to be highly expressed in solid tumors and is the member of the FOS family in activator protein (AP-1). Studies indicate that the AP-1 family members, including Fra-1, have essential effects in T cell development^45^. In melanoma, Fra-1 suppress the conversion of Treg cells into effector T cells under the regulation of Ubc13-IKK signaling axis^46^. In addition, Fra-1 is strongly associated with Epithelial-to-Mesenchymal Transition (EMT) in cancer cells^46,47^. It has been recently shown that EMT-related pathways could harm CD8 + T cell function, leading to immune evasion^48^. Moreover, a study from Lee et al. found a significant correlation between Fra-1 and PD-L1 expression, and high Fra-1 expression was associated with poorer overall survival^49^. Connexin 31 encoded by gap junction protein Beta 3 (GJB3), is one of the major members of the connexin family. Studies have indicated that connexin proteins mainly serve as channels to transport metabolites such as nucleotides, glutamate and glucose^50^. There is increasing evidence to support that metabolic changes in tumor cells affect the function of immune cells^52^. A study from Huo et al. reported that GJB3 promoted neutrophil survival and polarization by forming a channel between pancreatic tumor cells and neutrophils, transferring cyclic adenosine monophosphate (cAMP) from cancer to neutrophils^53^. Meanwhile, some studies suggested that cAMP could induce T-cell senescence^54,55^. Therefore, we hypothesized that GJB1 might be able to transfer cAMP to T cells in the same way which might cause T-cell exhaustion. The RNA modification N6-methyladenosine (m6A) has a significant value in the immune system^56^, and its dysregulation is associated with poor prognosis^57^. For example, a study showed that the m6A reader** i**nsulin-like growth factor-2 mRNA-binding protein 1 (IGF2BP1) could recognized the 30-UTR of PD-L1 mRNA and thus mediated stable PD-L1 expression^58^. The major histocompatibility complex, class II, DO beta (HLA-DOB) belonged to the HLA class II beta chain paralogue. It is demonstrated that HLA-II has antigen-presenting functions, participates in T-cell differentiation, and mediates the activation of T cells to provoke immune responses^59^. The insulin-like growth factor binding protein 1 (IGFBP1) has a vital action in regulating cell growth^60^. It had shown that ICFBP1 was expressed in T cells^61^. A research from Han et al. suggested that IGFBP1 had effects on CD4 + T cell immunomodulation^62^. Moreover, high expression of IGFBP1 is demonstrated to be closely associated with unfavorable OS in NSCLC^63^. Kallikrein related peptidase 11 (KLK11) is a member of the human KLK gene family, which is known to perform in a number of physiological processes, including extracellular matrix (ECM) remodeling, cell proliferation and differentiation^64^. Many studies have shown that KLK11 is aberrantly expressed in tumors and significantly correlated with survival^65–67^. The keratin 6A (KRT6A) and keratin 81 (KRT81) are the member of the keratin gene family. KRT6A gene overexpression in LUAD promotes lung cancer cell proliferation by EMT^68^. KRT81 has been identified as a promising biomarker for the identification of squamous cell lung cancer^69^.

The nine key genes described above may involved in T-cell exhaustion. Although the specific mechanisms have not yet been reported, we constructed a prognostic signature based on these genes that was confirmed to have some clinical significance. In the TCGA cohort, the prognostic signature was associated with OS, and patients in the low-risk group had a good prognosis. Then we performed external validation using the GSE72094 cohort to further confirm the general applicability of the prognostic signature. Moreover, we found that cluster 2 with high risk scores had the worst prognosis.

Immunotherapy had improved prognosis in lung cancer, but the benefit was limited. Only a minority of patients did benefit from ICIs^6^. One of the more widely utilized immune efficacy markers in clinical practice was PD-L1 expression. Numerous studies demonstrated that the expression level of PD-L1 is closely related to the efficacy of immunotherapy^70,71^. However, PD-L1 was not completely accurate in predicting immune efficacy. Hence, it was necessary to discover a signature that could predict the efficacy of immunotherapy. In our study, the prognostic signature we constructed may be useful to predict the immune response. Patients in the low-risk group had higher immune, stromal, and estimate scores, higher IPS, lower TIDE score which suggested a better response to immune checkpoint inhibitors (ICIs) therapy in the low-risk group. In the meantime, we included two separate immunotherapy cohorts, GSE135222 and GSE91061, which demonstrated better immune efficacy in the low-risk group. Besides, chemotherapy and targeted therapy were both the major strategies for the treatment of advanced LUAD. We found differences in the sensitivity of chemotherapeutic and targeted agents between high and low risk groups, suggesting that this prognostic signature might provide assistance in the selection of clinically sensitive agents for LUAD. Finally, the nomogram model combined with tumor stage and the riskscore could effectively predict the prognosis of LUAD patients.

Although our study had been successfully validated in an external cohort. However, there are several limitations in our study. Firstly, the study was based on publicly available databases and its training cohort (TCGA) and validation cohort (GEO) were retrospective. Therefore prospective studies are necessary to validate our conclusions. Secondly, there was a lack of molecular mechanism studies to investigate the functional role of candidate key genes, and in the future we will conduct further in vitro and in vivo experiments to confirm the potential regulatory mechanisms. Thirdly, in lack of a large cohort of lung cancer immunotherapy patients, our findings still need to be further validated by future prospective studies with larger samples.

## Conclusions

In summary, we identified nine key genes (including CPS1, FOSL1, GJB3, HLA-DOB, IGF2BP1, IGFBP1, KLK11, KRT6A, KRT81) that may involved in the regulation of T-cell exhaustion and constructed a riskscore that could help predict immunotherapy response and the selection of chemotherapeutic and targeted agents. In addition, the nomogram built in combination with tumor stage and the riskscore may be a powerful tool for LUAD survival prediction.

### Supplementary Information

Supplementary Tables.

Supplementary Figures.

## Data Availability

The datasets generated and analyzed during the current study are available in The Cancer Genome Atlas (TCGA) database (https://portal.gdc.cancer.gov/), and Gene Expression Omnibus (GEO) database (https://www.ncbi.nlm.nih.gov/). Access to both databases is not required accession number. The datasets analyzed during the current study are available from the corresponding author on reasonable request.

## References

[1] Siegel RL, Miller KD, Jemal A (2020). Cancer statistics, 2020. CA Cancer J. Clin..

[2] Dela Cruz CS, Tanoue LT, Matthay RA (2011). Lung cancer: Epidemiology, etiology, and prevention. Clin. Chest Med..

[3] Imielinski M, Berger AH, Hammerman PS, Hernandez B, Pugh TJ, Hodis E, Cho J, Suh J, Capelletti M, Sivachenko A (2012). Mapping the hallmarks of lung adenocarcinoma with massively parallel sequencing. Cell.

[4] Rittmeyer A, Barlesi F, Waterkamp D, Park K, Ciardiello F, von Pawel J, Gadgeel SM, Hida T, Kowalski DM, Dols MC (2017). Atezolizumab versus docetaxel in patients with previously treated non-small-cell lung cancer (OAK): A phase 3, open-label, multicentre randomised controlled trial. Lancet.

[5] Gettinger SN, Horn L, Gandhi L, Spigel DR, Antonia SJ, Rizvi NA, Powderly JD, Heist RS, Carvajal RD, Jackman DM (2015). Overall survival and long-term safety of nivolumab (anti-programmed death 1 antibody, BMS-936558, ONO-4538) in patients with previously treated advanced non-small-cell lung cancer. J. Clin. Oncol..

[6] Sui H, Ma N, Wang Y, Li H, Liu X, Su Y, Yang J (2018). Anti-PD-1/PD-L1 therapy for non-small-cell lung cancer: Toward personalized medicine and combination strategies. J. Immunol. Res..

[7] Jin MZ, Jin WL (2020). The updated landscape of tumor microenvironment and drug repurposing. Signal Transduct. Target Ther..

[8] Menares E, Galvez-Cancino F, Caceres-Morgado P, Ghorani E, Lopez E, Diaz X, Saavedra-Almarza J, Figueroa DA, Roa E, Quezada SA (2019). Tissue-resident memory CD8(+) T cells amplify anti-tumor immunity by triggering antigen spreading through dendritic cells. Nat. Commun..

[9] Hanahan D, Coussens LM (2012). Accessories to the crime: Functions of cells recruited to the tumor microenvironment. Cancer Cell.

[10] Bremnes RM, Busund L-T, Kilvær TL, Andersen S, Richardsen E, Paulsen EE, Hald S, Khanehkenari MR, Cooper WA, Kao SC (2016). The role of tumor-infiltrating lymphocytes in development, progression, and prognosis of non-small cell lung cancer. J. Thorac. Oncol..

[11] Thommen DS, Schumacher TN (2018). T cell dysfunction in cancer. Cancer Cell.

[12] Franco F, Jaccard A, Romero P, Yu YR, Ho PC (2020). Metabolic and epigenetic regulation of T-cell exhaustion. Nat. Metab..

[13] McLane LM, Abdel-Hakeem MS, Wherry EJ (2019). CD8 T cell exhaustion during chronic viral infection and cancer. Annu. Rev. Immunol..

[14] Wherry EJ, Kurachi M (2015). Molecular and cellular insights into T cell exhaustion. Nat. Rev. Immunol..

[15] Boyero L, Sanchez-Gastaldo A, Alonso M, Noguera-Ucles JF, Molina-Pinelo S, Bernabe-Caro R (2020). Primary and acquired resistance to immunotherapy in lung cancer: Unveiling the mechanisms underlying of immune checkpoint blockade therapy. Cancers (Basel).

[16] Langfelder P, Horvath S (2008). WGCNA: An R package for weighted correlation network analysis. BMC Bioinform..

[17] Wilkerson MD, Hayes DN (2010). ConsensusClusterPlus: A class discovery tool with confidence assessments and item tracking. Bioinformatics.

[18] Yu GWL, Han Y, He QY (2012). clusterProfiler: An R package for comparing biological themes among gene clusters. OMICS.

[19] Liberzon ABC, Thorvaldsdóttir H, Ghandi M, Mesirov JP, Tamayo P (2015). The molecular signatures database (MSigDB) hallmark gene set collection. Cell Syst..

[20] Subramanian ATP, Mootha VK, Mukherjee S, Ebert BL, Gillette MA, Paulovich A, Pomeroy SL, Golub TR, Lander ES, Mesirov JP (2005). Gene set enrichment analysis: A knowledge-based approach for interpreting genome-wide expression profiles. Proc. Natl. Acad. Sci. U. S. A..

[21] Yoshihara K, Shahmoradgoli M, Martinez E, Vegesna R, Kim H, Torres-Garcia W, Trevino V, Shen H, Laird PW, Levine DA (2013). Inferring tumour purity and stromal and immune cell admixture from expression data. Nat. Commun..

[22] Newman AM, Liu CL, Green MR, Gentles AJ, Feng W, Xu Y, Hoang CD, Diehn M, Alizadeh AA (2015). Robust enumeration of cell subsets from tissue expression profiles. Nat. Methods.

[23] Miao YR, Zhang Q, Lei Q, Luo M, Xie GY, Wang H, Guo AY (2020). ImmuCellAI: A unique method for comprehensive T-cell subsets abundance prediction and its application in cancer immunotherapy. Adv. Sci. (Weinh.).

[24] Love MI, Huber W, Anders S (2014). Moderated estimation of fold change and dispersion for RNA-seq data with DESeq2. Genome Biol..

[25] Tibshirani R (1997). The lasso method for variable selection in the Cox model. Stat. Med..

[26] Taylor JM (2011). Random survival forests. J. Thorac. Oncol..

[27] Jiang P, Gu S, Pan D, Fu J, Sahu A, Hu X, Li Z, Traugh N, Bu X, Li B (2018). Signatures of T cell dysfunction and exclusion predict cancer immunotherapy response. Nat. Med..

[28] Charoentong P, Finotello F, Angelova M, Mayer C, Efremova M, Rieder D, Hackl H, Trajanoski Z (2017). Pan-cancer immunogenomic analyses reveal genotype-immunophenotype relationships and predictors of response to checkpoint blockade. Cell Rep..

[29] Chan TAYM, Jaffee E, Swanton C, Quezada SA, Stenzinger A, Peters S (2019). Development of tumor mutation burden as an immunotherapy biomarker: Utility for the oncology clinic. Ann. Oncol..

[30] Mayakonda A, Lin DC, Assenov Y, Plass C, Koeffler HP (2018). Maftools: Efficient and comprehensive analysis of somatic variants in cancer. Genome Res..

[31] Geeleher P, Cox N, Huang RS (2014). pRRophetic: An R package for prediction of clinical chemotherapeutic response from tumor gene expression levels. PLoS ONE.

[32] Wang Z, Wu X (2020). Study and analysis of antitumor resistance mechanism of PD1/PD-L1 immune checkpoint blocker. Cancer Med..

[33] Schalper KA, Brown J, Carvajal-Hausdorf D, McLaughlin J, Velcheti V, Syrigos KN, Herbst RS, Rimm DL (2015). Objective measurement and clinical significance of TILs in non-small cell lung cancer. J. Natl. Cancer Inst..

[34] Al-Shibli KI, Donnem T, Al-Saad S, Persson M, Bremnes RM, Busund LT (2008). Prognostic effect of epithelial and stromal lymphocyte infiltration in non-small cell lung cancer. Clin. Cancer Res..

[35] Xiang Z, Zhou Z, Song S, Li J, Ji J, Yan R, Wang J, Cai W, Hu W, Zang L (2021). Dexamethasone suppresses immune evasion by inducing GR/STAT3 mediated downregulation of PD-L1 and IDO1 pathways. Oncogene.

[36] Arumuggam N, Bhowmick NA, Rupasinghe HP (2015). A review: Phytochemicals targeting JAK/STAT signaling and IDO expression in cancer. Phytother. Res..

[37] Qian J, Wang C, Wang B, Yang J, Wang Y, Luo F, Xu J, Zhao C, Liu R, Chu Y (2018). The IFN-gamma/PD-L1 axis between T cells and tumor microenvironment: Hints for glioma anti-PD-1/PD-L1 therapy. J. Neuroinflamm..

[38] Ju X, Zhang H, Zhou Z, Chen M, Wang Q (2020). Tumor-associated macrophages induce PD-L1 expression in gastric cancer cells through IL-6 and TNF-a signaling. Exp. Cell Res..

[39] Morris SM (2002). Regulation of enzymes of the urea cycle and arginine metabolism. Annu. Rev. Nutr..

[40] Tarasenko TN, Gomez-Rodriguez J, McGuire PJ (2015). Impaired T cell function in argininosuccinate synthetase deficiency. J. Leukoc. Biol..

[41] Ochoa JB, Strange J, Kearney P, Gellin G, Endean E, Fitzpatrick E (2001). Effects of L-arginine on the proliferation of T lymphocyte subpopulations. JPEN J. Parenter Enteral. Nutr..

[42] Zea AH, Rodriguez PC, Culotta KS, Hernandez CP, DeSalvo J, Ochoa JB, Park HJ, Zabaleta J, Ochoa AC (2004). L-Arginine modulates CD3zeta expression and T cell function in activated human T lymphocytes. Cell Immunol..

[43] Mussai F, Egan S, Hunter S, Webber H, Fisher J, Wheat R, McConville C, Sbirkov Y, Wheeler K, Bendle G (2015). Neuroblastoma arginase activity creates an immunosuppressive microenvironment that impairs autologous and engineered immunity. Cancer Res..

[44] Kosenko E, Venediktova N, Kaminsky Y, Montoliu C, Felipo V (2003). Sources of oxygen radicals in brain in acute ammonia intoxication in vivo. Brain Res..

[45] Jochum WPE, Wagner EF (2001). AP-1 in mouse development and tumorigenesis. Oncogene.

[46] Chang JHXY, Hu H, Jin J, Yu J, Zhou X, Wu X, Johnson HM, Akira S, Pasparakis M, Cheng X, Sun SC (2012). Ubc13 maintains the suppressive function of regulatory T cells and prevents their conversion into effector-like T cells. Nat. Immunol..

[47] Dhillon AS, Tulchinsky E (2015). FRA-1 as a driver of tumour heterogeneity: A nexus between oncogenes and embryonic signalling pathways in cancer. Oncogene.

[48] Talotta F, Casalino L, Verde P (2020). The nuclear oncoprotein Fra-1: A transcription factor knocking on therapeutic applications' door. Oncogene.

[49] Chai AWY, Lim KP, Cheong SC (2020). Translational genomics and recent advances in oral squamous cell carcinoma. Semin. Cancer Biol..

[50] Lee MHYJ, Tran L, Walser TC, Bisht B, Fung E, Park SJ, Zeng G, Krysan K, Wallace WD, Paul MK, Girard L, Gao B, Minna JD, Dubinett SM, Lee JM (2020). FRA1 contributes to MEK-ERK pathway-dependent PD-L1 upregulation by KRAS mutation in premalignant human bronchial epithelial cells. Am. J. Transl. Res..

[51] Sanchez A, Castro C, Flores DL, Gutierrez E, Baldi P (2019). Gap junction channels of innexins and connexins: Relations and computational perspectives. Int. J. Mol. Sci..

[52] Chang CH, Qiu J, O'Sullivan D, Buck MD, Noguchi T, Curtis JD, Chen Q, Gindin M, Gubin MM, van der Windt GJ (2015). Metabolic competition in the tumor microenvironment is a driver of cancer progression. Cell.

[53] Huo Y, Zhou Y, Zheng J, Jin G, Tao L, Yao H, Zhang J, Sun Y, Liu Y, Hu LP (2022). GJB3 promotes pancreatic cancer liver metastasis by enhancing the polarization and survival of neutrophil. Front Immunol..

[54] Ye J, Peng G (2015). Controlling T cell senescence in the tumor microenvironment for tumor immunotherapy. Oncoimmunology.

[55] Ye J, Ma C, Hsueh EC, Dou J, Mo W, Liu S, Han B, Huang Y, Zhang Y, Varvares MA (2014). TLR8 signaling enhances tumor immunity by preventing tumor-induced T-cell senescence. EMBO Mol. Med..

[56] Shulman Z, Stern-Ginossar N (2020). The RNA modification N(6)-methyladenosine as a novel regulator of the immune system. Nat. Immunol..

[57] Huang H, Weng H, Chen J (2020). m(6)A modification in coding and non-coding RNAs: Roles and therapeutic implications in cancer. Cancer Cell.

[58] Ni Z, Sun P, Zheng J, Wu M, Yang C, Cheng M, Yin M, Cui C, Wang G, Yuan L (2022). JNK signaling promotes bladder cancer immune escape by regulating METTL3-mediated m6A modification of PD-L1 mRNA. Cancer Res..

[59] Adams EJ, Luoma AM (2013). The adaptable major histocompatibility complex (MHC) fold: Structure and function of nonclassical and MHC class I-like molecules. Annu. Rev. Immunol..

[60] Baxter RC (2014). IGF binding proteins in cancer: Mechanistic and clinical insights. Nat. Rev. Cancer.

[61] Uhlen M, Fagerberg L, Hallstrom BM, Lindskog C, Oksvold P, Mardinoglu A, Sivertsson A, Kampf C, Sjostedt E, Asplund A (2015). Proteomics. Tissue-based map of the human proteome. Science.

[62] Han K, Singh K, Rodman MJ, Hassanzadeh S, Baumer Y, Huffstutler RD, Chen J, Candia J, Cheung F, Stagliano KER (2021). Identification and validation of nutrient state-dependent serum protein mediators of human CD4(+) T cell responsiveness. Nutrients.

[63] Wang J, Hu ZG, Li D, Xu JX, Zeng ZG (2019). Gene expression and prognosis of insulin-like growth factor-binding protein family members in non-small cell lung cancer. Oncol. Rep..

[64] Borgono CA, Diamandis EP (2004). The emerging roles of human tissue kallikreins in cancer. Nat. Rev. Cancer.

[65] Sasaki H, Kawano O, Endo K, Suzuki E, Haneda H, Yukiue H, Kobayashi Y, Yano M, Fujii Y (2006). Decreased kallikrein 11 messenger RNA expression in lung cancer. Clin. Lung Cancer.

[66] Patsis C, Yiotakis I, Scorilas A (2012). Diagnostic and prognostic significance of human kallikrein 11 (KLK11) mRNA expression levels in patients with laryngeal cancer. Clin. Biochem..

[67] Kolin DL, Sy K, Rotondo F, Bassily MN, Kovacs K, Brezden-Masley C, Streutker CJ, Yousef GM (2016). Prognostic significance of human tissue kallikrein-related peptidases 11 and 15 in gastric cancer. Tumour Biol..

[68] Yang BZW, Zhang M, Wang X, Peng S, Zhang R (2020). KRT6A promotes EMT and cancer stem cell transformation in lung adenocarcinoma. Technol. Cancer Res. Treat..

[69] Campayo M, Navarro A, Vinolas N, Tejero R, Munoz C, Diaz T, Marrades R, Cabanas ML, Gimferrer JM, Gascon P (2011). A dual role for KRT81: A miR-SNP associated with recurrence in non-small-cell lung cancer and a novel marker of squamous cell lung carcinoma. PLoS ONE.

[70] Garon EB, Hellmann MD, Rizvi NA, Carcereny E, Leighl NB, Ahn MJ, Eder JP, Balmanoukian AS, Aggarwal C, Horn L (2019). Five-year overall survival for patients with advanced non-small-cell lung cancer treated with pembrolizumab: Results from the phase I KEYNOTE-001 study. J. Clin. Oncol..

[71] Borghaei H, Paz-Ares L, Horn L, Spigel DR, Steins M, Ready NE, Chow LQ, Vokes EE, Felip E, Holgado E (2015). Nivolumab versus docetaxel in advanced nonsquamous non-small-cell lung cancer. N. Engl. J. Med..

