# Supplementary material for: The identification of genes associated T-cell exhaustion and construction of prognostic signature to predict immunotherapy response in lung adenocarcinoma
Source: Sci Rep. 2023 Aug 17;13:13415. doi: 10.1038/s41598-023-40662-z (PMC10435542; doi:10.1038/s41598-023-40662-z)
Supplement: Supplementary file 1 — Supplementary Tables. [file 41598_2023_40662_MOESM1_ESM.pdf]

Table S1. The clinical features of TCGA-LUAD and GSE72094 cohorts.

Characteristic	TCGA-LUAD (training dataset N=539)	GSE72094 (validation dataset N=442)
age		
≤65	257 (47.7%)	127 (28.7%)
>65	263 (48.8%)	294 (66.5%)
unknown	19 (3.5%)	21 (4.8%)
gender		
female	289 (53.6%)	240 (54.3%)
male	250 (46.4%)	202 (45.7%)
T stage		
T1	176 (32.7%)	-
T2	292 (54.2%)	-
T3	49 (9.1%)	-
T4	19 (3.5%)	-
unknown	3 (0.6%)	-
N stage		
N0	350 (64.9%)	-
N1	97 (18%)	-
N2	74 (13.7%)	-
N3	2 (0.4%)	-
unknown	16 (3%)	-
M stage		
M0	365 (67.7%)	-
M1	25 (4.6%)	-
unknown	149 (27.6%)	-
Pathological stage		
I	296 (54.9%)	265 (60%)
II	125 (23.2%)	69 (15.6%)
III	84 (15.6%)	63 (14.3%)
IV	26 (4.8%)	17 (3.8%)
unknown	8 (1.5%)	28 (6.3%)

Table S2 The information of GEO datasets.

**Dataset**	**Platform**	**Sequencing type**
TCGA-LUAD	Illumina HiSeq2000 RNA sequencing platform	Expression profiling by high throughput sequencing
GSE72094	GPL15048 Rosetta/Merck Human RSTA Custom Affymetrix 2.0 microarray	Expression profiling by array
GSE135222	GPL16791 Illumina HiSeq 2500	Expression profiling by high throughput sequencing
GSE91061	GPL9052 Illumina Genome Analyzer	Expression profiling by high throughput sequencing
