# Supplementary material for: The identification of genes associated T-cell exhaustion and construction of prognostic signature to predict immunotherapy response in lung adenocarcinoma
Source: Sci Rep. 2023 Aug 17;13:13415. doi: 10.1038/s41598-023-40662-z (PMC10435542; doi:10.1038/s41598-023-40662-z)
Supplement: Supplementary file 2 — Supplementary Figures. [file 41598_2023_40662_MOESM2_ESM.pdf]

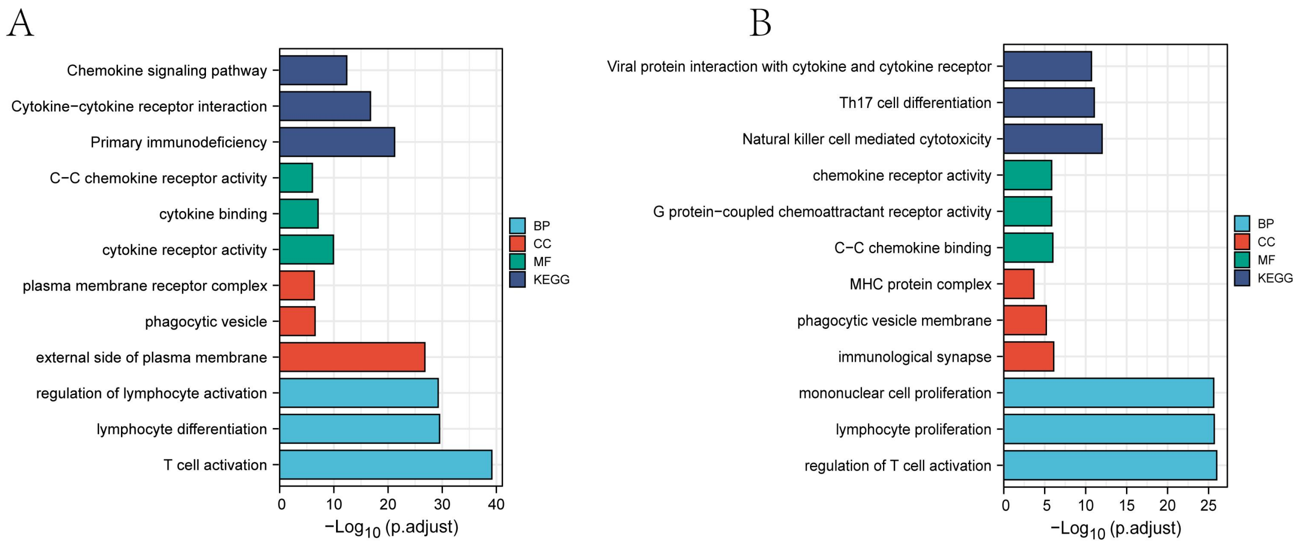

Figure S1. Gene Ontology (GO) and Kyoto Encyclopedia of Genes and Genomes (KEGG) analysis were performed with T-cell exhaustion related genes.

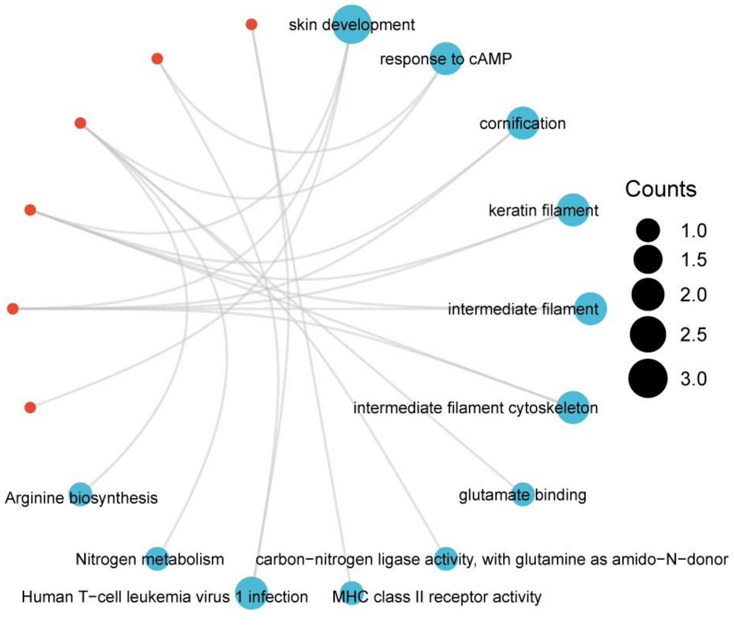

Figure S2. Gene Ontology (GO) and Kyoto Encyclopedia of Genes and Genomes (KEGG) analysis based on 9 hub genes associated with regulation of T-cell exhaustion.

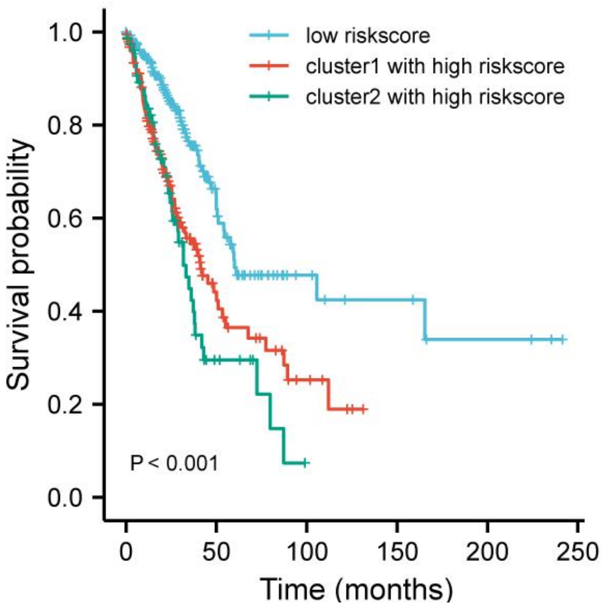

Figure S3. Kaplan-Meier survival analysis for patients in different subgroup.

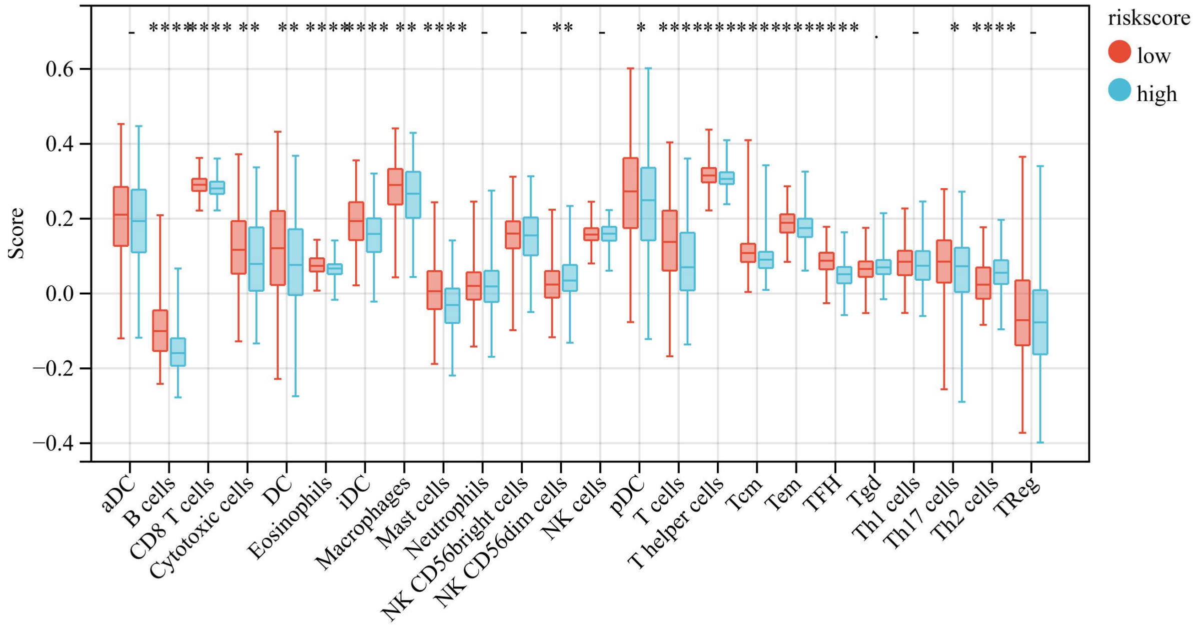

Figure S4. Single sample gene set enrichment analysis (ssGSEA)for immune cell infiltration scores in different risk groups.

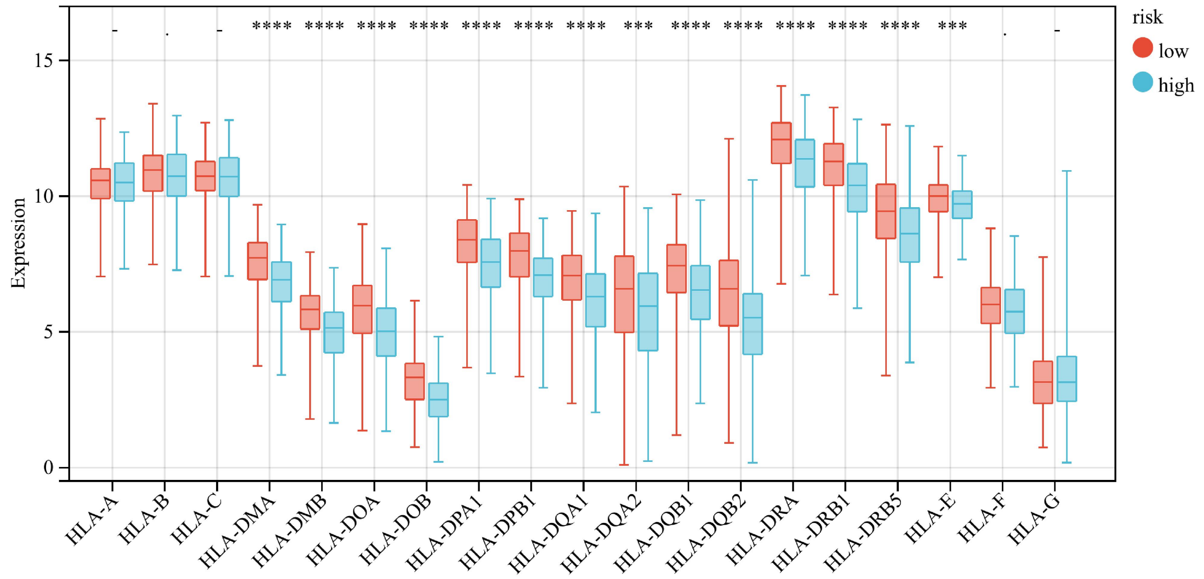

Figure S5. The distribution of the mRNA expression of HLA gene sets in different risk groups.
